# Cystatin C Is a Predictor for Long-Term, All-Cause, and Cardiovascular Mortality in US Adults With Metabolic Syndrome

**DOI:** 10.1210/clinem/dgae225

**Published:** 2024-04-10

**Authors:** Xiaoying Song, Lijiao Xiong, Tianting Guo, Xue Chen, Pinjun Zhang, Xiaoan Zhang, Zhen Liang

**Affiliations:** Medical Department, Ganzhou People's Hospital, Ganzhou, Jiangxi Province, 341000, China; Department of Geriatrics, Shenzhen People's Hospital (The Second Clinical Medical College, Jinan University; The First Affiliated Hospital, Southern University of Science and Technology), Shenzhen, 518000, China; Ganzhou Hospital of Guangdong Provincial People's Hospital (Ganzhou Municipal Hospital), Ganzhou, Jiangxi Province, 341000, China; Department of Geriatrics, Shenzhen People's Hospital (The Second Clinical Medical College, Jinan University; The First Affiliated Hospital, Southern University of Science and Technology), Shenzhen, 518000, China; Guangdong Provincial Clinical Research Center for Geriatrics, Shenzhen Clinical Research Center for Geriatrics, Shenzhen People's Hospital (The Second Clinical Medical College, Jinan University; The First Affiliated Hospital, Southern University of Science and Technology), Shenzhen, 518000, China; Gannan Medical University, Ganzhou, Jiangxi Province, 341000, China; Ganzhou Hospital of Guangdong Provincial People's Hospital (Ganzhou Municipal Hospital), Ganzhou, Jiangxi Province, 341000, China; Department of Geriatrics, Shenzhen People's Hospital (The Second Clinical Medical College, Jinan University; The First Affiliated Hospital, Southern University of Science and Technology), Shenzhen, 518000, China; Guangdong Provincial Clinical Research Center for Geriatrics, Shenzhen Clinical Research Center for Geriatrics, Shenzhen People's Hospital (The Second Clinical Medical College, Jinan University; The First Affiliated Hospital, Southern University of Science and Technology), Shenzhen, 518000, China

**Keywords:** Cystatin C, metabolic syndrome, all-cause mortality, CVD mortality, cancer mortality, prognostic marker

## Abstract

**Objective:**

This study examined the relationship between cystatin C (CysC) levels and all-cause, cardiovascular disease (CVD), and cancer mortality in US metabolic syndrome (MetS) patients.

**Methods:**

The 1999-2002 National Health and Nutrition Examination Survey (NHANES) prospective cohort research included 1980 MetS participants. To assess CysC levels and all-cause, CVD, and cancer mortality, fitted curves, Kaplan-Meier survival curves, Cox regression analysis, and receiver operating characteristic curves were performed.

**Results:**

During a mean follow-up of 15.3 ± 5.4 years, a total of 819 deaths occurred. The fitted and Kaplan-Meier survival curves revealed that greater CysC levels were linked to higher all-cause, CVD, and cancer mortality rates (*P* < .05). After adjusting for variables, CysC level was associated with all-cause, CVD, and cancer mortality at 1.63 (1.42-1.88), 1.53 (1.19-1.95), and 1.53 (1 ∼ 2.32), respectively (*P* < .05). Tertile models showed consistent results: high CysC Tertile participants showed higher risk of all-cause mortality (HR 1.87; 1.43-2.45), CVD mortality (HR 1.97, 1.15 ∼ 3.38), and cancer mortality (HR 1.72, 1.01 ∼ 2.91) compared to those in the lowest tertile (*P* < .05). Subgroup studies by sex and other characteristics confirmed the findings. CysC demonstrated the higher predictive efficacy across mortality outcomes, followed by eGFR, outperforming urea nitrogen, creatinine, uric acid, and C-reactive protein. CysC alone exhibited substantial predictive value for all-cause (AUC 0.773; *P* < .05) and CVD mortality (AUC 0.726; *P* < .05). Combining CysC with age enhanced predictive value for all-cause mortality to 0.861 and CVD mortality to 0.771 (*P* < .05).

**Conclusion:**

MetS patients with elevated CysC levels have a higher risk of all-cause, CVD, and cancer death. CysC may predict MetS all-cause and CVD mortality.

Metabolic syndrome (MetS), characterized by central obesity, elevated blood sugar, hypertension, and abnormal lipid levels, has seen a significant uptick in prevalence, currently affecting an estimated 34.7% of US adults (1). This surge raises concerns due to its close link with cardiovascular disease (CVD) and heightened mortality risk. Those diagnosed with MetS not only face an elevated susceptibility to developing CVD, but also bear a risk of reduced life expectancy due to a higher likelihood of experiencing severe CVD events such as heart attacks and strokes (2, 3). Additionally, they are at an increased vulnerability to other chronic conditions, including type 2 diabetes, heart disease, and specific types of cancer, all contributing to a decreased overall life expectancy (2-4). Furthermore, research has consistently shown that individuals with MetS have an increased likelihood of developing various types of cancer, such as breast, colorectal, endometrial, pancreatic, gastric, and prostate cancer, among others (5, 6). Moreover, those with MetS who are diagnosed with cancer tend to experience a higher risk of mortality (5).

Cystatin C (CysC), an endogenous marker of renal function, has garnered attention as a potential prognostic biomarker in various disease states (7). In addition to its role in estimating glomerular filtration rate, CysC has garnered attention for its potential to reflect broader pathophysiological processes beyond kidney function (7, 8). Research suggests that elevated levels of CysC are associated with conditions such as inflammation, oxidative stress, and endothelial dysfunction—factors that are not only relevant to kidney disease, but also play crucial roles in the development and progression of various CVD and metabolic disorders, including MetS (9-12). Despite this mechanistic plausibility, the prognostic significance of CysC in individuals with MetS remains underexplored, presenting a critical knowledge gap in risk stratification and personalized care, particularly regarding long-term mortality prediction.

This study seeks to address this gap by examining the relationship between CysC levels and mortality risks in individuals with MetS, leveraging a well-characterized longitudinal cohort with extended follow-up. By investigating the independent association of CysC with all-cause, CVD, and cancer mortality in this high-risk population, we aim to elucidate its potential as a robust prognostic marker. Furthermore, we endeavor to explore potential effect modifiers and interactions, such as age, gender, and comorbidity burden, to inform tailored risk assessment and targeted interventions.

## Methods

### Data Sources and Preparation

The data utilized in this study were obtained from the National Health and Nutrition Examination Survey (NHANES), a prospective cohort research initiative. NHANES is a nationally representative survey of noninstitutionalized individuals in the United States and involves participants who provided written informed consent following approval by the NCHS institutional review board. Employing a multilevel, stratified probability design, the survey annually samples 5000 participants who undergo standardized questionnaires and physical examinations. Data collection has been ongoing since 1999, with updated datasets released biennially at https://www.cdc.gov/nchs/nhanes/index.htm. The research protocol was reviewed by the institutional review board at Shenzhen People's Hospital, which determined that the study did not necessitate informed consent as it involved publicly available, de-identified data.

The NHANES 1999-2002 data with 21 004 individuals were utilized in this study, which were restricted by specific exclusion criteria. The population in the present study remains consistent with that of our previous study (13). A total of 10 768 individuals were excluded due to missing CysC data, 161 due to missing height data, and 80 due to missing weight data. Additionally, exclusions were made for missing marital status (364), education (8), alcohol consumption (353), tobacco use (8), annual family income (223), telomere length (2528), chronic kidney disease (32), heart attack (7), congestive heart failure (15), coronary heart disease (22), hypertension (1), angina (18), stroke (4), anemia (2), mortality (3), and non-MetS (4427). Ultimately, 1980 individuals with MetS were enrolled in this study.

### Metabolic Syndrome

As per the criteria established by the International Diabetes Federation Task Force on Epidemiology and Prevention; National Heart, Lung, and Blood Institute; American Heart Association; World Heart Federation; International Atherosclerosis Society; and the International Association for the Study of Obesity, the diagnosis of MetS requires the presence of at least 3 of the following 5 conditions (14): These criteria encompass an increased abdominal obesity (waist circumference ≥ 88 cm for female individuals and ≥ 102 cm for male individuals), elevated triglyceride levels(≥ 150 mg/dL or receiving medication), reduced high-density lipoprotein-cholesterol (HDL-C) levels (< 40 mg/dL for men and < 50 mg/dL for women or receiving medication), elevated blood pressure (systolic blood pressure ≥ 130 mmHg or diastolic blood pressure ≥ 85 mmHg or on antihypertensive medication), and elevated fasting plasma glucose levels (≥ 100 mg/dL or receiving medication) (14, 15).

### Serum Laboratory Parameters

The Dade Behring N Latex CysC assay was used to measure CysC levels in blood samples (16). This assessment was conducted on the Dade Behring Nephelometer II. According to Newman's evaluation of various assay methodologies, this current assay is deemed the most accurate and precise among automated assays across the clinical concentration range, demonstrating an intra-assay imprecision range between 2.0% and 3.0% coefficient of variation. The inter-assay imprecision range falls between 3.2 and 4.4% coefficient of variation. Furthermore, the assay range spans from 0.23 to 7.25 mg/dL. Newman also observed that this assay exhibited superior sensitivity and lacked analytical interference when compared to other automated assays (16). Serum albumin, globulin, fast glucose, fast insulin, creatinine, uric acid, blood urea nitrogen, triglyceride, total cholesterol, HDL-C, low-density lipoprotein-cholesterol (LDL-C), and C-reactive protein (CRP) were analyzed by laboratory methods reported elsewhere (17) Serum creatinine levels were measured using the kinetic Jaffe rate method (18). Albumin levels were detected using the Bromcresol Purple method within the Boehringer Mannheim Diagnostics albumin system (17). Cholesterol levels were detected through an enzymatic reaction coupled with photometry (17). Glucose levels were assessed using the glucose hexokinase method, while insulin levels were quantified using the insulin radioimmunoassay (RIA) (17). Blood urea nitrogen levels were determined using the enzymatic kinetic method, while uric acid levels were assessed through the colorimetric method. CRP levels were quantified using latex-enhanced nephelometry (17).

### All-Cause, Cardiovascular, and Cancer Mortality

The study focused on all-cause mortality, CVD mortality, and cancer mortality as the primary outcomes. All-cause mortality referred to the number of participants who passed away due to any cause after completing their baseline survey but before December 31, 2018. Mortality follow-up data was obtained from NHANES Public-Use Linked Mortality Files (https://www.cdc.gov/nchs/data-linkage/mortality.htm) using the International Classification of Diseases, Tenth Revision (ICD-10) codes to identify causes of death. These alphanumeric codes are used to track diseases and health-related issues, enabling researchers to categorize deaths based on the leading causes of death. Specifically, CVD deaths were identified using specific ICD codes (054-068), encompassing coronary artery disease, heart failure, stroke, and peripheral artery disease. This method ensured accurate tracking of deaths related to CVD by utilizing precise ICD codes for categorization (19, 20).

### Covariates

Our study encompassed a range of clinical and demographic factors as covariates to address potential sources of confounding. These covariate variables included age, sex, body mass index (BMI), race and ethnicity, educational level, marital status, smoking status, alcohol drinking status, annual family income, and chronic diseases. Information regarding these covariates was derived from survey responses in NHANES. Participants were classified into 5 racial/ethnic groups: Mexican American, other Hispanic, non-Hispanic White, Black, or Other (including multiracial). Educational classifications comprised less than high school, high school graduate or equivalent, some college or AA degree, and college graduate or above (21). Marital status was delineated using the 3 categories: Never married; Married; and Widowed, divorced, or separated. Medical conditions such as anemia, angina, heart attack, congestive heart failure, coronary heart disease, chronic kidney disease, asthma, chronic obstructive pulmonary disease (COPD), diabetes mellitus (DM), hypertension, hyperlipidemia (22), cancer, and stroke were diagnosed by a physician or other qualified healthcare professional. Smoking and drinking behaviors were grouped into 3 categories: never, past, and current use. BMI was calculated using the standard formula: weight (kg)/[height (m) × height (m)]. The calculation of estimated glomerular filtration rate (eGFR) was performed using the CKD-EPI equation (23).

## Statistical Analysis

Continuous variables were accompanied by 95% CIs, while categorical variables were presented as percentage frequencies. Comparison of continuous and categorical data was performed using *t* tests and χ^2^ tests. Given the low rates of missing data for all variables, no imputation approach was employed. Mortality risk was assessed using Cox proportional hazards regression models. We employed Cox regression models with the following adjustments: no adjustment (Model 1); adjusted for age, sex, and BMI (Model 2); Model 2 with additional adjustments for race, marital status, education, alcohol use, smoking status, annual family income, and eGFR (Model 3); and Model 3 with further adjustments for asthma, congestive heart failure, coronary heart disease, chronic kidney disease, COPD, DM, hypertension, hyperlipidemia, stroke, and cancer (Model 4). Visual representation was provided through curve fitting and Kaplan-Meier curves. The statistical analyses were conducted using the R software package (http://www.R-project.org, The R Foundation), the nhanesR package, and Free Statistics software version 1.9. Statistical significance was determined by a two-sided *P* value < .05.

## Results

### Demographics

Table 1 presents the characteristics of 1980 individuals with MetS, divided into 3 CysC tertiles. The average age was 58.0 ± 16.5 years, with significant age group variations. Gender distribution showed a 51% female and 49% male split. Race composition varied significantly. Differences in alcohol use, smoking history, marital status, and education were observed, with significant income disparities. CysC Tertile 3 (CysC T3) individuals were distinctively older (mean age 68.0 ± 14.2 years) and predominantly male (51.7%) (Table 1). They had a higher proportion of non-Hispanic whites (65%), former alcohol users (30.2%), and were less likely to be current smokers (15.7%). CysC T3 displayed lower estimated kidney function, with higher rates of chronic conditions, including congestive heart failure (8%), coronary heart disease (9%), DM (27.4%), hypertension (81%), hyperlipidemia (83.7%), stroke (6.9%), and cancer (18.6%) (Table 1). In terms of medication usage, individuals in CysC T3 demonstrated a notably higher utilization of hypertension and high cholesterol medications (Supplementary Table S1) (24).

**Table 1.** Baseline characteristics of participants with metabolic syndrome in NHANES 1999-2002

Characteristics	Total (n = 1980)	CysC Tertile 1 (n = 658)	CysC Tertile 2 (n = 659)	CysC Tertile 3 (n = 663)	*P* value
Cystatin C (mg/L), mean ± SD	0.90 ± 0.42	0.67 ± 0.07	0.82 ± 0.04	1.20 ± 0.61	<.001
Age (years), mean ± SD	58.0 ± 16.5	48.7 ± 14.3	57.1 ± 14.9	68.0 ± 14.2	<.001
Age group, n (%)					<.001
< 45 years	434 (21.9)	253 (38.4)	128 (19.4)	53 (8)	
45-64 years	775 (39.1)	309 (47)	299 (45.4)	167 (25.2)	
≥ 65 years	771 (38.9)	96 (14.6)	232 (35.2)	443 (66.8)	
Gender, n (%)					<.001
Female	1009 (51.0)	379 (57.6)	310 (47)	320 (48.3)	
Male	971 (49.0)	279 (42.4)	349 (53)	343 (51.7)	
Height (cm), mean ± SD	167.2 ± 10.3	166.5 ± 9.8	168.1 ± 10.5	166.9 ± 10.6	.011
Weight (kg), mean ± SD	86.8 ± 19.8	85.7 ± 18.4	88.3 ± 19.8	86.5 ± 21.0	.053
BMI (kg/m^2^), mean ± SD	31.0 ± 5.9	30.8 ± 5.7	31.1 ± 5.8	30.9 ± 6.3	.682
Race, n (%)					<.001
Mexican American	438 (22.1)	202 (30.7)	146 (22.2)	90 (13.6)	
Non-Hispanic Black	335 (16.9)	131 (19.9)	93 (14.1)	111 (16.7)	
Non-Hispanic White	1075 (54.3)	272 (41.3)	372 (56.4)	431 (65)	
Other Hispanic	87 (4.4)	30 (4.6)	35 (5.3)	22 (3.3)	
Other race—including multiracial	45 (2.3)	23 (3.5)	13 (2)	9 (1.4)	
Alcohol user, n (%)					<.001
Former	483 (24.4)	118 (17.9)	165 (25)	200 (30.2)	
Heavy	324 (16.4)	160 (24.3)	110 (16.7)	54 (8.1)	
Mild	646 (32.6)	206 (31.3)	212 (32.2)	228 (34.4)	
Moderate	209 (10.6)	89 (13.5)	79 (12)	41 (6.2)	
Never	318 (16.1)	85 (12.9)	93 (14.1)	140 (21.1)	
Smoking, n (%)					.039
Former	704 (35.6)	216 (32.8)	245 (37.2)	243 (36.7)	
Never	958 (48.4)	348 (52.9)	294 (44.6)	316 (47.7)	
Now	318 (16.1)	94 (14.3)	120 (18.2)	104 (15.7)	
Marital status, n (%)					<.001
Divorced or separated or widowed	584 (29.5)	156 (23.7)	176 (26.7)	252 (38)	
Married	1249 (63.1)	423 (64.3)	442 (67.1)	384 (57.9)	
Never married	147 (7.4)	79 (12)	41 (6.2)	27 (4.1)	
Education, n (%)					.077
College graduate or above	351 (17.7)	121 (18.4)	117 (17.8)	113 (17)	
High school grad/GED or equivalent	475 (24.0)	143 (21.7)	158 (24)	174 (26.2)	
Less than high school	691 (34.9)	220 (33.4)	225 (34.1)	246 (37.1)	
Some college or AA degree	463 (23.4)	174 (26.4)	159 (24.1)	130 (19.6)	
Annual family income, n (%)					<.001
$0 to $19 999	644 (32.5)	176 (26.7)	197 (29.9)	271 (40.9)	
$20 000 to $34 999	488 (24.6)	140 (21.3)	179 (27.2)	169 (25.5)	
$35 000 to $54 999	327 (16.5)	124 (18.8)	105 (15.9)	98 (14.8)	
more than $55 000	521 (26.3)	218 (33.1)	178 (27)	125 (18.9)	
eGFR (mL/min), mean ± SD	86.1 ± 24.2	102.9 ± 18.0	88.6 ± 16.8	67.0 ± 22.2	<.001
Anemia, n (%)					<.001
No anemia	1859 (93.9)	623 (94.7)	640 (97.1)	596 (89.9)	
Mild	90 (4.5)	25 (3.8)	16 (2.4)	49 (7.4)	
Moderate	30 (1.5)	10 (1.5)	3 (0.5)	17 (2.6)	
Severe	1 (0.1)	0 (0)	0 (0)	1 (0.2)	
Asthma, n (%)					.231
No	1696 (85.7)	566 (86)	574 (87.1)	556 (83.9)	
Yes	284 (14.3)	92 (14)	85 (12.9)	107 (16.1)	
Chronic kidney disease, n (%)					<.001
No	1463 (73.9)	573 (87.1)	549 (83.3)	341 (51.4)	
Yes	517 (26.1)	85 (12.9)	110 (16.7)	322 (48.6)	
Heart attack, n (%)					<.001
No	1868 (94.3)	644 (97.9)	633 (96.1)	591 (89.1)	
Yes	112 (5.7)	14 (2.1)	26 (3.9)	72 (10.9)	
Congestive heart failure, n (%)					<.001
No	1895 (95.7)	647 (98.3)	638 (96.8)	610 (92)	
Yes	85 (4.3)	11 (1.7)	21 (3.2)	53 (8)	
COPD, n (%)					<.001
No	1918 (96.9)	652 (99.1)	636 (96.5)	630 (95)	
Yes	62 (3.1)	6 (0.9)	23 (3.5)	33 (5)	
Coronary heart disease, n (%)					<.001
No	1873 (94.6)	641 (97.4)	629 (95.4)	603 (91)	
Yes	107 (5.4)	17 (2.6)	30 (4.6)	60 (9)	
DM, n (%)					<.001
No	1244 (66.0)	430 (71.2)	426 (67.1)	388 (60.1)	
DM	436 (23.1)	126 (20.9)	133 (20.9)	177 (27.4)	
IFG	205 (10.9)	48 (7.9)	76 (12)	81 (12.5)	
Hypertension, n (%)					<.001
No	601 (30.4)	273 (41.5)	202 (30.7)	126 (19)	
Yes	1379 (69.6)	385 (58.5)	457 (69.3)	537 (81)	
Hyperlipidemia, n (%)					.072
No	291 (14.7)	103 (15.7)	80 (12.1)	108 (16.3)	
Yes	1689 (85.3)	555 (84.3)	579 (87.9)	555 (83.7)	
Stroke, n (%)					<.001
No	1905 (96.2)	652 (99.1)	636 (96.5)	617 (93.1)	
Yes	75 (3.8)	6 (0.9)	23 (3.5)	46 (6.9)	
Cancer, n (%)					<.001
No	1741 (87.9)	622 (94.5)	579 (87.9)	540 (81.4)	
Yes	239 (12.1)	36 (5.5)	80 (12.1)	123 (18.6)	
All-cause mortality, n (%)					<.001
Alive	1161 (58.6)	546 (83)	413 (62.7)	202 (30.5)	
Dead	819 (41.4)	112 (17)	246 (37.3)	461 (69.5)	
Albumin (g/L), mean ± SD	43.4 ± 3.4	43.6 ± 3.5	43.7 ± 3.2	42.8 ± 3.3	<.001
Globulin (g/L), mean ± SD	31.7 ± 4.3	31.1 ± 3.9	31.7 ± 4.2	32.3 ± 4.8	.006
Fasting glucose (mg/dL), mean ± SD	117.11 ± 46.22	116.72 ± 51.51	114.96 ± 37.63	119.69 ± 48.33	.271
Fasting insulin (pmol/L), mean ± SD	107.14 ± 123.82	103.24 ± 132.18	101.20 ± 80.47	117.11 ± 148.37	.092
Creatinine (mg/dL), mean ± SD	0.88 ± 0.60	0.70 ± 0.19	0.81 ± 0.20	1.12 ± 0.94	<.001
Uric acid (mg/dL), Mean ± SD	5.73 ± 1.51	5.13 ± 1.40	5.73 ± 1.39	6.34 ± 1.51	<.001
Blood urea nitrogen (mg/dL), mean ± SD	15.25 ± 6.28	12.63 ± 4.05	14.45 ± 4.10	18.65 ± 8.11	<.001
Triglyceride (mg/dL), mean ± SD	190.98 ± 147.51	185.31 ± 196.46	198.46 ± 119.40	189.21 ± 110.31	.356
Total cholesterol (mg/dL), mean ± SD	215.95 ± 40.92	216.81 ± 40.51	217.97 ± 40.10	213.11 ± 42.01	.078
HDL-C (mg/dL), mean ± SD	52.23 ± 15.09	54.61 ± 16.05	51.07 ± 14.56	51.02 ± 14.34	<.001
LDL-C (mg/dL), mean ± SD	129.08 ± 35.44	130.88 ± 36.09	128.93 ± 34.35	127.30 ± 35.84	.333
C-reactive protein (mg/dL), mean ± SD	0.55 ± 0.75	0.53 ± 0.68	0.51 ± 0.73	0.60 ± 0.82	.07
Death cause, n (%)					.388
Accidents	25 (3.1)	4 (3.6)	10 (4.1)	11 (2.4)	
Alzheimer disease	34 (4.2)	5 (4.5)	9 (3.7)	20 (4.3)	
Cerebrovascular diseases	44 (5.4)	5 (4.5)	18 (7.3)	21 (4.6)	
Chronic lower respiratory diseases	33 (4.0)	4 (3.6)	8 (3.3)	21 (4.6)	
Diabetes mellitus	33 (4.0)	5 (4.5)	11 (4.5)	17 (3.7)	
Diseases of heart	231 (28.2)	25 (22.3)	62 (25.2)	144 (31.2)	
Influenza and pneumonia	11 (1.3)	0 (0)	6 (2.4)	5 (1.1)	
Cancer	183 (22.3)	32 (28.6)	59 (24)	92 (20)	
Nephritis, nephrotic syndrome, and nephrosis	12 (1.5)	0 (0)	5 (2)	7 (1.5)	
All other causes	213 (26.0)	32 (28.6)	58 (23.6)	123 (26.7)	
Follow-up time (years), mean ± SD	15.3 ± 5.4	17.7 ± 3.3	16.1 ± 4.8	12.2 ± 6.0	<.001

Abbreviations: BMI, body mass index; COPD, chronic obstructive pulmonary disease; CysC, cystatin C; DM, diabetes mellitus; eGFR, estimated glomerular filtration rate; HDL-C, high-density lipoprotein-cholesterol; IFG, impaired fasting glucose; NHANES, National Health and Nutrition Examination Survey.

Elevated CysC tertiles correlate with a progressive decline in serum albumin levels (*P* < .001) and a concomitant rise in globulin concentrations (*P* = .006), suggesting potential renal filtration impairment and altered protein metabolism as CysC levels increase (Table 1). While fasting glucose (*P* = .271), insulin (*P* = .092), triglycerides (*P* = .356), total cholesterol (*P* = .078), and LDL cholesterol (*P* = .333) exhibit no significant trends across CysC tertiles, there is a notable escalation in serum creatinine (*P* < .001), uric acid (*P* < .001), and blood urea nitrogen (*P* < .001) levels with higher CysC tertiles, indicative of deteriorating renal function (Table 1). Moreover, individuals in higher CysC tertiles demonstrate a decline in HDL-C levels (*P* < .001), underscoring an adverse impact on lipid metabolism associated with renal dysfunction (Table 1). CRP levels display marginal significance (*P* = .07) across CysC tertiles, failing to meet the predetermined threshold for statistical significance (Table 1).

The study followed the participants for an average duration of 15.3 ± 5.4 years. The follow-up period differed significantly among the CysC tertiles (*P* < .001). CysC T1 had the longest mean follow-up of 17.7 ± 3.3 years, while CysC T2 and T3 had mean follow-up periods of 16.1 ± 4.8 years and 12.2 ± 6.0 years, respectively (Table 1). Throughout the follow-up period, the study observed a total of 819 (41.4%) deaths. The distribution of causes of death displayed no statistically significant differences overall (*P* = .388). The causes of death included accidents (3.1%), Alzheimer disease (4.2%), cerebrovascular diseases (5.4%), chronic lower respiratory diseases (4.0%), DM (4.0%), diseases of the heart (28.2%), influenza and pneumonia (1.3%), cancer (22.3%), nephritis, nephrotic syndrome, and nephrosis (1.5%), and all other causes (26.0%) (Table 1). Patients with MetS showed significant differences in mortality rates based on their use of antihypertensive and cholesterol-lowering medications (*P* < .05) (Supplementary Table S2) (24). Specifically, those using these medications had higher mortality rates compared to nonusers (Supplementary Figs. S1 and S2) (24).

### Hazard Ratios for All-Cause, CVD, and Cancer Mortality Stratified by CysC Levels in Participants With MetS

**Figure 1.**
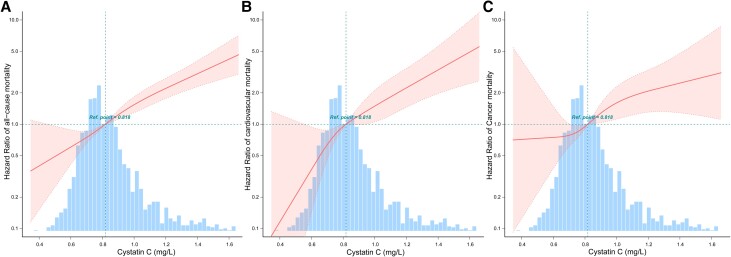
The relationship between cystatin C (CysC) and all-cause, CVD, and cancer mortality by curve fitting. Adjusted for age, BMI, race, education level, marriage status, drinking, smoking, annual family income, and eGFR. (A) The curve fitting of CysC and all-cause mortality in metabolic syndrome participants. (B) The curve fitting of CysC and CVD mortality in metabolic syndrome participants. (C) The curve fitting of CysC and cancer mortality in metabolic syndrome participants.

**Figure 2.**
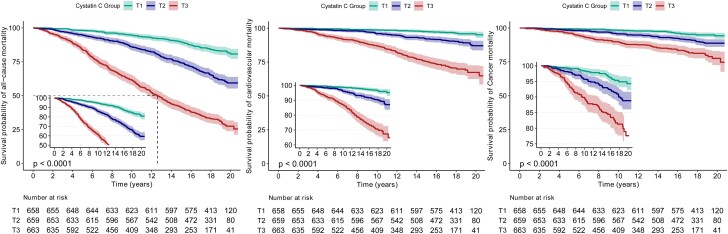
Kaplan-Meier survival curves for CysC associated with all-cause, CVD, and cancer mortality risk. (A) The Kaplan-Meier survival curves of CysC and all-cause mortality in metabolic syndrome participants. (B) The Kaplan-Meier survival curves of CysC and CVD mortality in metabolic syndrome participants. (C) The Kaplan-Meier survival curves of CysC and cancer mortality in metabolic syndrome participants.

The fitted curves indicated that lower CysC levels in participants with MetS decreased all-cause mortality (Fig. 1A), CVD mortality (Fig. 1B), and cancer mortality (Fig. 1C). Conversely, as CysC levels increased, the risk of all-cause, CVD mortality, and cancer mortality increased significantly (Fig. 1A-1C). Kaplan-Meier survival curves also suggested that higher CysC levels were associated with increased all-cause mortality, CVD mortality, and cancer mortality risk (*P* < .05) (Fig. 2A-2C). After adjusted for various covariates in multiple models, the hazard ratio (HR) of total CysC level with all-cause, CVD, and cancer mortality were 1.63 (95% CI, 1.42-1.88; *P* < .001), 1.53 (95% CI, 1.19-1.95; *P* = .001), and 1.53 (95% CI, 1 ∼ 2.32; *P* = .048), respectively (Table 2). The HRs stratified by CysC levels for all-cause, CVD, and cancer mortality in participants with MetS were adjusted for various covariates in multiple models.

**Table 2.** The hazard ratios of CysC with all-cause, cardiovascular, and cancer mortality in the participants with metabolic syndrome

Variable	Total	Event (%)	Model 1	*P* value	Model 2	*P* value	Model 3	*P* value	Model 4	*P* value
**All-cause mortality**										
CysC total	1980	819 (41.4)	1.86 (1.74 ∼ 1.99)	<.001	1.75 (1.6 ∼ 1.92)	<.001	1.7 (1.49 ∼ 1.95)	<.001	1.63 (1.42 ∼ 1.88)	<.001
CysC Tertile 1	658	112 (17)	1 (Ref)		1 (Ref)		1 (Ref)		1 (Ref)	
CysC Tertile 2	659	246 (37.3)	2.5 (2 ∼ 3.12)	<.001	1.5 (1.19 ∼ 1.89)	.001	1.31 (1.04 ∼ 1.66)	.024	1.37 (1.08 ∼ 1.74)	.01
CysC Tertile 3	663	461 (69.5)	6.71 (5.45 ∼ 8.26)	<.001	2.39 (1.9 ∼ 3.01)	<.001	1.88 (1.45 ∼ 2.44)	<.001	1.87 (1.43 ∼ 2.45)	<.001
Trend test	1980	819 (41.4)	2.61 (2.37 ∼ 2.88)	<.001	1.56 (1.4 ∼ 1.74)	<.001	1.38 (1.22 ∼ 1.57)	<.001	1.37 (1.2 ∼ 1.56)	<.001
**Cardiovascular mortality**										
CysC total	1980	231 (11.7)	1.95 (1.74 ∼ 2.18)	<.001	1.92 (1.65 ∼ 2.23)	<.001	1.69 (1.34 ∼ 2.13)	<.001	1.53 (1.19 ∼ 1.95)	.001
CysC Tertile 1	658	25 (3.8)	1 (Ref)		1 (Ref)		1 (Ref)		1 (Ref)	
CysC Tertile 2	659	62 (9.4)	2.82 (1.77 ∼ 4.48)	<.001	1.56 (0.97 ∼ 2.51)	.066	1.28 (0.79 ∼ 2.08)	.323	1.42 (0.87 ∼ 2.31)	.163
CysC Tertile 3	663	144 (21.7)	9.35 (6.1 ∼ 14.32)	<.001	2.84 (1.78 ∼ 4.54)	<.001	1.89 (1.11 ∼ 3.2)	.018	1.97 (1.15 ∼ 3.38)	.014
Trend test	1980	231 (11.7)	3.14 (2.59 ∼ 3.8)	<.001	1.73 (1.39 ∼ 2.14)	<.001	1.4 (1.09 ∼ 1.8)	.009	1.4 (1.08 ∼ 1.81)	.01
**Cancer mortality**										
CysC total	1980	183 (9.2)	1.65 (1.38 ∼ 1.97)	<.001	1.44 (1.09 ∼ 1.9)	.011	1.46 (1 ∼ 2.14)	.052	1.52 (1 ∼ 2.32)	.049
CysC Tertile 1	658	32 (4.9)	1 (Ref)		1 (Ref)		1 (Ref)		1 (Ref)	
CysC Tertile 2	659	59 (9)	2.09 (1.36 ∼ 3.21)	.001	1.38 (0.89 ∼ 2.16)	.151	1.25 (0.79 ∼ 1.97)	.346	1.19 (0.75 ∼ 1.89)	.464
CysC Tertile 3	663	92 (13.9)	4.58 (3.05 ∼ 6.86)	<.001	2.01 (1.28 ∼ 3.18)	.003	1.84 (1.1 ∼ 3.09)	.021	1.72 (1.01 ∼ 2.91)	.045
Trend test	1980	183 (9.2)	2.15 (1.77 ∼ 2.61)	<.001	1.42 (1.14 ∼ 1.78)	.002	1.38 (1.06 ∼ 1.78)	.015	1.33 (1.02 ∼ 1.73)	.033

Abbreviation: CysC, cystatin C.

Model 1: Crude Model.

Model 3: Adjusted for age, sex, and body mass index.

Model 3: Adjusted for age, sex, race, marital status, body mass index, education, alcohol user, smoking status, annual family income and estimated glomerular filtration rate (eGFR).

Model 4: Adjusted for age, sex, race, marital status, body mass index, education, alcohol user, smoking status, annual family income, eGFR, asthma, congestive heart failure, coronary heart disease, chronic kidney disease, chronic obstructive pulmonary disease (COPD), diabetes mellitus, hypertension, hyperlipidemia, stroke, and cancer.

### All-Cause Mortality

Among the study participants, 819 individuals (41.4%) died of all causes. Higher CysC levels were consistently associated with increased mortality risk in all models. In the crude model (Model 1), the HR was 1.86 (95% CI, 1.74-1.99; *P* < .001), and this association persisted in subsequent models. In Model 4, which was most comprehensive in covariate adjustment, the HR was 1.63 (95% CI, 1.42-1.88; *P* < .001). A clear dose-response relationship was observed with CysC Tertile 3 displaying the highest HR (1.87; 95% CI, 1.43-2.45; *P* < .001), confirming a substantial increase in mortality risk with elevated CysC levels. The trend test revealed a dose-response relationship with an HR of 1.37 (95% CI, 1.2-1.56; *P* < .001) (Table 2).

### Cardiovascular Mortality

Out of the total participants, 231 (11.7%) died of CVD causes. Similar to all-cause mortality, higher CysC levels were significantly associated with elevated CVD mortality risk across all models. Model 1 showed a HR of 1.95 (95% CI, 1.74-2.18; *P* < .001), with consistent associations in subsequent models. In Model 4, the HR was 1.53 (95% CI, 1.19-1.95; *P* < .001). CysC Tertile 3 exhibited the highest HR (1.97; 95% CI, 1.15 ∼ 3.38; *P* = .014), indicating the greatest risk of CVD mortality. The trend test confirmed the dose-response relationship with an HR of 1.4 (95% CI, 1.08-1.81; *P* = .01) (Table 2).

### Cancer Mortality

Within the cohort, 183 participants (9.2%) died of cancer-related causes. While Model 1 demonstrated a significant association between CysC levels and cancer mortality (HR 1.65; 95% CI, 1.38-1.97; *P* < .001), this significance varied across models. In Models 2 and 4, the association re-emerged (*P* = .011 and *P* = .049, respectively). When stratified by CysC tertiles, CysC Tertile 3 displayed the highest HR (HR 1.72; 95% CI, 1.01 ∼ 2.91; *P* = .045), indicative of a strong association with cancer mortality, and a dose-response relationship was observed (*P* for trend < .001) (Table 2).

### Covariate Adjustment

In Models 2, 3, and 4, various covariates were systematically adjusted, progressively refining the analysis. Model 4, which comprehensively accounted for demographic factors, lifestyle variables, eGFR, and a spectrum of comorbidities, underscored the robust, independent, and dose-dependent association between elevated CysC levels and increased all-cause, CVD, and cancer mortality in individuals with MetS. These results provided compelling evidence for the significant impact of CysC levels on mortality outcomes, even after meticulous adjustments for multiple confounding factors in Model 4, further emphasizing the critical role of CysC as a prognostic marker in individuals with MetS.

### Subgroup Analyses

To investigate the robustness of the association between CysC and all-cause mortality, CVD mortality, and cancer mortality in individuals with MetS, stratified analyses were conducted across various subgroups. The examined variables included gender, asthma, congestive heart failure, coronary heart disease, chronic kidney disease, COPD, DM, hypertension, hyperlipidemia, stroke, and cancer. However, none of these variables demonstrated a significant modification of the relationship between CysC and the risk of all-cause mortality, CVD mortality, or cancer mortality in individuals with MetS (all *P* values for interaction > .05), indicating an absence of substantive impact on the association between CysC levels and mortality risks by these factors (Table 3).

**Table 3.** The HRs of CysC with all-cause mortality or cardiovascular mortality in the participants with metabolic syndrome in subgroup analyses

Subgroup	Total	All-cause mortality				Cardiovascular mortality				Cancer mortality			
		All-cause deaths (%)	adj. HR	adj. *P* value	*P* for interaction	CVD deaths (%)	adj. HR	adj. *P* value	*P* for interaction	Cancer deaths (%)	adj. HR	adj. *P* value	*P* for interaction
			(95% CI)				(95% CI)				(95% CI)		
**Gender = Female**					.259				.159				.705
CysC Tertile 1	379	65 (17.2)	1 (Ref)			16 (4.2)	1 (Ref)			16 (4.2)	1 (Ref)		
CysC Tertile 2	310	110 (35.5)	1.1 (0.78 ∼ 1.53)	.589		24 (7.7)	0.77 (0.38 ∼ 1.55)	.46		26 (8.4)	1.31 (0.67 ∼ 2.58)	.428	
CysC Tertile 3	320	208 (65)	1.82 (1.26 ∼ 2.64)	.001		58 (18.1)	1.35 (0.63 ∼ 2.89)	.441		42 (13.1)	2.45 (1.15 ∼ 5.2)	.02	
Trend test	1009	383 (38)	1.4 (1.17 ∼ 1.69)	<.001		98 (9.7)	1.26 (0.86 ∼ 1.85)	.241		84 (8.3)	1.61 (1.1 ∼ 2.34)	.014	
**Gender = Male**													
CysC Tertile 1	279	47 (16.8)	1 (Ref)			9 (3.2)	1 (Ref)			16 (5.7)	1 (Ref)		
CysC Tertile 2	349	136 (39)	1.59 (1.12 ∼ 2.24)	.009		38 (10.9)	2.11 (1 ∼ 4.46)	.05		33 (9.5)	1.22 (0.65 ∼ 2.29)	.542	
CysC Tertile 3	343	253 (73.8)	2.06 (1.4 ∼ 3.01)	<.001		86 (25.1)	2.81 (1.27 ∼ 6.22)	.011		50 (14.6)	1.51 (0.73 ∼ 3.12)	.269	
Trend test	971	436 (44.9)	1.4 (1.17 ∼ 1.67)	<.001		133 (13.7)	1.55 (1.1 ∼ 2.17)	.012		99 (10.2)	1.23 (0.86 ∼ 1.76)	.258	
**Asthma = no**					.252				.325				.318
CysC Tertile 1	566	91 (16.1)	1 (Ref)			18 (3.2)	1 (Ref)			27 (4.8)	1 (Ref)		
CysC Tertile 2	574	208 (36.2)	1.35 (1.04 ∼ 1.75)	.023		56 (9.8)	1.64 (0.94 ∼ 2.85)	.081		49 (8.5)	1.28 (0.78 ∼ 2.11)	.335	
CysC Tertile 3	556	392 (70.5)	2.05 (1.54 ∼ 2.74)	<.001		122 (21.9)	2.53 (1.38 ∼ 4.6)	.003		80 (14.4)	2.21 (1.26 ∼ 3.87)	.006	
Trend test	1696	691 (40.7)	1.45 (1.26 ∼ 1.67)	<.001		196 (11.6)	1.57 (1.19 ∼ 2.07)	.001		156 (9.2)	1.53 (1.15 ∼ 2.02)	.003	
**Asthma = yes**													
CysC Tertile 1	92	21 (22.8)	1 (Ref)			7 (7.6)	1 (Ref)			5 (5.4)	1 (Ref)		
CysC Tertile 2	85	38 (44.7)	1.05 (0.58 ∼ 1.9)	.859		6 (7.1)	0.31 (0.09 ∼ 1.13)	.076		10 (11.8)	1.06 (0.31 ∼ 3.63)	.924	
CysC Tertile 3	107	69 (64.5)	1.03 (0.53 ∼ 1.97)	.937		22 (20.6)	0.45 (0.12 ∼ 1.64)	.227		12 (11.2)	0.6 (0.14 ∼ 2.54)	.485	
Trend test	284	128 (45.1)	1.01 (0.73 ∼ 1.39)	.957		35 (12.3)	0.74 (0.38 ∼ 1.44)	.373		27 (9.5)	0.76 (0.38 ∼ 1.52)	.434	
**CKD = no**					.775				.545				.671
CysC Tertile 1	573	91 (15.9)	1 (Ref)			21 (3.7)	1 (Ref)			27 (4.7)	1 (Ref)		
CysC Tertile 2	549	184 (33.5)	1.46 (1.11 ∼ 1.92)	.007		49 (8.9)	1.36 (0.78 ∼ 2.38)	.278		50 (9.1)	1.4 (0.84 ∼ 2.34)	.196	
CysC Tertile 3	341	195 (57.2)	2.14 (1.57 ∼ 2.92)	<.001		54 (15.8)	1.73 (0.91 ∼ 3.26)	.093		48 (14.1)	1.91 (1.05 ∼ 3.45)	.033	
Trend test	1463	470 (32.1)	1.46 (1.26 ∼ 1.7)	<.001		124 (8.5)	1.3 (0.96 ∼ 1.77)	.09		125 (8.5)	1.38 (1.03 ∼ 1.85)	.031	
CKD = yes													
CysC Tertile 1	85	21 (24.7)	1 (Ref)			4 (4.7)	1 (Ref)			5 (5.9)	1 (Ref)		
CysC Tertile 2	110	62 (56.4)	1.39 (0.82 ∼ 2.37)	.224		13 (11.8)	1.27 (0.39 ∼ 4.17)	.694		9 (8.2)	1.11 (0.34 ∼ 3.61)	.861	
CysC Tertile 3	322	266 (82.6)	1.74 (0.99 ∼ 3.05)	.053		90 (28)	2.27 (0.67 ∼ 7.64)	.185		44 (13.7)	2 (0.59 ∼ 6.81)	.269	
Trend test	517	349 (67.5)	1.3 (1.01 ∼ 1.66)	.043		107 (20.7)	1.63 (0.96 ∼ 2.75)	.069		58 (11.2)	1.5 (0.83 ∼ 2.72)	.177	
**Congestive heart failure = no**					.213				.144				.849
CysC Tertile 1	647	110 (17)	1 (Ref)			24 (3.7)	1 (Ref)			31 (4.8)	1 (Ref)		
CysC Tertile 2	638	230 (36.1)	1.28 (1.01 ∼ 1.63)	.043		54 (8.5)	1.15 (0.69 ∼ 1.91)	.583		58 (9.1)	1.27 (0.8 ∼ 2.02)	.312	
CysC Tertile 3	610	416 (68.2)	1.9 (1.46 ∼ 2.49)	<.001		126 (20.7)	1.82 (1.05 ∼ 3.15)	.032		89 (14.6)	1.86 (1.1 ∼ 3.15)	.02	
Trend test	1895	756 (39.9)	1.4 (1.23 ∼ 1.6)	<.001		204 (10.8)	1.41 (1.08 ∼ 1.84)	.012		178 (9.4)	1.38 (1.07 ∼ 1.79)	.015	
**Congestive heart failure = yes**													
CysC Tertile 1	11	2 (18.2)	1 (Ref)			1 (9.1)	1 (Ref)			1 (9.1)	1 (Ref)		
CysC Tertile 2	21	16 (76.2)	21.62 (3.48 ∼ 134.14)	.001		8 (38.1)	30.36 (2.09 ∼ 441.29)	.012		1 (4.8)	6.09 (0 ∼ Inf)	.999	
CysC Tertile 3	53	45 (84.9)	12.55 (1.91 ∼ 82.56)	.009		18 (34)	11.36 (0.96 ∼ 134.01)	.054		3 (5.7)	4.08 (0 ∼ Inf)	.998	
Trend test	85	63 (74.1)	1.75 (0.9 ∼ 3.41)	.098		27 (31.8)	1.93 (0.81 ∼ 4.63)	.138		5 (5.9)	4.08 (0 ∼ Inf)	.965	
**COPD = no**					.432				.118				.458
CysC Tertile 1	652	109 (16.7)	1 (Ref)			23 (3.5)	1 (Ref)			32 (4.9)	1 (Ref)		
CysC Tertile 2	636	229 (36)	1.29 (1.02 ∼ 1.65)	.036		57 (9)	1.36 (0.82 ∼ 2.26)	.236		56 (8.8)	1.25 (0.79 ∼ 1.98)	.349	
CysC Tertile 3	630	432 (68.6)	1.89 (1.45 ∼ 2.47)	<.001		135 (21.4)	2.2 (1.27 ∼ 3.8)	.005		85 (13.5)	1.81 (1.07 ∼ 3.07)	.028	
Trend test	1918	770 (40.1)	1.39 (1.22 ∼ 1.59)	<.001		215 (11.2)	1.52 (1.17 ∼ 1.97)	.002		173 (9)	1.36 (1.05 ∼ 1.77)	.021	
**COPD = yes**													
CysC Tertile 1	6	3 (50)	1 (Ref)			2 (33.3)	1 (Ref)			0 (0)	1 (Ref)		
CysC Tertile 2	23	17 (73.9)	4.18 (0.74 ∼ 23.75)	.106		5 (21.7)	0.15 (0.04 ∼ 0.49)	.002		3 (13)	—	.999	
CysC Tertile 3	33	29 (87.9)	2.37 (0.32 ∼ 17.68)	.401		9 (27.3)	0.02 (0.01 ∼ 0.07)	<.001		7 (21.2)	—	.999	
Trend test	62	49 (79)	0.91 (0.4 ∼ 2.08)	.83		16 (25.8)	0.15 (0.07 ∼ 0.33)	<.001		10 (16.1)	—	.226	
**Coronary heart disease = no**					.6				.079				.203
CysC Tertile 1	641	105 (16.4)	1 (Ref)			24 (3.7)	1 (Ref)			29 (4.5)	1 (Ref)		
CysC Tertile 2	629	228 (36.2)	1.31 (1.03 ∼ 1.68)	.029		51 (8.1)	1.16 (0.69 ∼ 1.94)	.57		58 (9.2)	1.37 (0.85 ∼ 2.2)	.194	
CysC Tertile 3	603	409 (67.8)	1.93 (1.47 ∼ 2.53)	<.001		118 (19.6)	1.92 (1.1 ∼ 3.34)	.022		86 (14.3)	1.98 (1.16 ∼ 3.38)	.012	
Trend test	1873	742 (39.6)	1.4 (1.23 ∼ 1.6)	<.001		193 (10.3)	1.45 (1.11 ∼ 1.9)	.007		173 (9.2)	1.41 (1.09 ∼ 1.84)	.01	
**Coronary heart disease = yes**													
CysC Tertile 1	17	7 (41.2)	1 (Ref)			1 (5.9)	1 (Ref)			3 (17.6)	1 (Ref)		
CysC Tertile 2	30	18 (60)	1.05 (0.35 ∼ 3.11)	.936		11 (36.7)	2.37 (0.25 ∼ 22.42)	.451		1 (3.3)	0 (0 ∼ 0.01)	<.001	
CysC Tertile 3	60	52 (86.7)	1.55 (0.51 ∼ 4.7)	.435		26 (43.3)	2.41 (0.25 ∼ 23.28)	.449		6 (10)	0.02 (0.01 ∼ 0.09)	<.001	
Trend test	107	77 (72)	1.28 (0.75 ∼ 2.21)	.368		38 (35.5)	1.3 (0.54 ∼ 3.09)	.557		10 (9.3)	0.47 (0.04 ∼ 6.11)	.563	
**DM = DM**					.105				.321				.498
CysC Tertile 1	126	33 (26.2)	1 (Ref)			9 (7.1)	1 (Ref)			10 (7.9)	1 (Ref)		
CysC Tertile 2	133	83 (62.4)	1.79 (1.16 ∼ 2.75)	.008		26 (19.5)	2.07 (0.91 ∼ 4.68)	.081		11 (8.3)	0.78 (0.3 ∼ 2.04)	.614	
CysC Tertile 3	177	137 (77.4)	1.73 (1.04 ∼ 2.87)	.034		47 (26.6)	1.98 (0.77 ∼ 5.13)	.157		16 (9)	0.73 (0.23 ∼ 2.36)	.599	
Trend test	436	253 (58)	1.26 (0.99 ∼ 1.6)	.061		82 (18.8)	1.3 (0.84 ∼ 2.02)	.233		37 (8.5)	0.86 (0.47 ∼ 1.55)	.605	
**DM = IFG**													
CysC Tertile 1	48	14 (29.2)	1 (Ref)			3 (6.2)	1 (Ref)			5 (10.4)	1 (Ref)		
CysC Tertile 2	76	30 (39.5)	1.09 (0.52 ∼ 2.26)	.822		6 (7.9)	0.91 (0.18 ∼ 4.5)	.909		10 (13.2)	1.38 (0.37 ∼ 5.1)	.628	
CysC Tertile 3	81	59 (72.8)	1.38 (0.62 ∼ 3.07)	.436		16 (19.8)	2 (0.38 ∼ 10.58)	.413		16 (19.8)	1.35 (0.3 ∼ 6.05)	.693	
Trend test	205	103 (50.2)	1.19 (0.8 ∼ 1.76)	.383		25 (12.2)	1.56 (0.68 ∼ 3.57)	.294		31 (15.1)	1.13 (0.55 ∼ 2.32)	.737	
**DM = no**													
CysC Tertile 1	430	63 (14.7)	1 (Ref)			13 (3)	1 (Ref)			17 (4)	1 (Ref)		
CysC Tertile 2	426	130 (30.5)	1.15 (0.83 ∼ 1.59)	.398		30 (7)	1.13 (0.56 ∼ 2.27)	.731		37 (8.7)	1.57 (0.85 ∼ 2.9)	.15	
CysC Tertile 3	388	265 (68.3)	2.03 (1.43 ∼ 2.89)	<.001		81 (20.9)	1.95 (0.93 ∼ 4.07)	.075		60 (15.5)	2.73 (1.38 ∼ 5.4)	.004	
Trend test	1244	458 (36.8)	1.5 (1.27 ∼ 1.79)	<.001		124 (10)	1.49 (1.05 ∼ 2.12)	.026		114 (9.2)	1.67 (1.2 ∼ 2.33)	.002	
**Hypertension = no**					.8				.975				.274
CysC Tertile 1	273	29 (10.6)	1 (Ref)			6 (2.2)	1 (Ref)			7 (2.6)	1 (Ref)		
CysC Tertile 2	202	52 (25.7)	1.49 (0.91 ∼ 2.45)	.116		11 (5.4)	1.27 (0.41 ∼ 3.92)	.682		19 (9.4)	2.54 (0.97 ∼ 6.61)	.057	
CysC Tertile 3	126	67 (53.2)	2.32 (1.31 ∼ 4.1)	.004		15 (11.9)	2.16 (0.59 ∼ 7.94)	.244		16 (12.7)	2.95 (0.97 ∼ 9.01)	.057	
Trend test	601	148 (24.6)	1.53 (1.15 ∼ 2.02)	.003		32 (5.3)	1.51 (0.79 ∼ 2.88)	.213		42 (7)	1.61 (0.96 ∼ 2.69)	.07	
**Hypertension = yes**													
CysC Tertile 1	385	83 (21.6)	1 (Ref)			19 (4.9)	1 (Ref)			25 (6.5)	1 (Ref)		
CysC Tertile 2	457	194 (42.5)	1.3 (0.99 ∼ 1.7)	.06		51 (11.2)	1.34 (0.77 ∼ 2.32)	.294		40 (8.8)	1.07 (0.63 ∼ 1.81)	.809	
CysC Tertile 3	537	394 (73.4)	1.84 (1.37 ∼ 2.48)	<.001		129 (24)	1.98 (1.1 ∼ 3.57)	.023		76 (14.2)	1.71 (0.94 ∼ 3.09)	.077	
Trend test	1379	671 (48.7)	1.37 (1.19 ∼ 1.58)	<.001		199 (14.4)	1.43 (1.08 ∼ 1.88)	.012		141 (10.2)	1.35 (1 ∼ 1.81)	.051	
**Hyperlipidemia = no**					.417				.139				.865
CysC Tertile 1	103	18 (17.5)	1 (Ref)			2 (1.9)	1 (Ref)			8 (7.8)	1 (Ref)		
CysC Tertile 2	80	28 (35)	1.11 (0.57 ∼ 2.15)	.763		5 (6.2)	1.92 (0.31 ∼ 12.03)	.487		8 (10)	1.54 (0.5 ∼ 4.73)	.452	
CysC Tertile 3	108	84 (77.8)	2.32 (1.19 ∼ 4.51)	.013		28 (25.9)	7.99 (1.34 ∼ 47.65)	.023		18 (16.7)	3.18 (1.02 ∼ 9.86)	.045	
Trend test	291	130 (44.7)	1.64 (1.19 ∼ 2.27)	.003		35 (12)	3.31 (1.49 ∼ 7.37)	.003		34 (11.7)	1.82 (1.05 ∼ 3.18)	.034	
**Hyperlipidemia = yes**													
CysC Tertile 1	555	94 (16.9)	1 (Ref)			23 (4.1)	1 (Ref)			24 (4.3)	1 (Ref)		
CysC Tertile 2	579	218 (37.7)	1.32 (1.02 ∼ 1.7)	.036		57 (9.8)	1.21 (0.73 ∼ 2.02)	.457		51 (8.8)	1.32 (0.79 ∼ 2.21)	.291	
CysC Tertile 3	555	377 (67.9)	1.79 (1.34 ∼ 2.39)	<.001		116 (20.9)	1.51 (0.86 ∼ 2.64)	.152		74 (13.3)	1.79 (0.99 ∼ 3.23)	.053	
Trend test	1689	689 (40.8)	1.34 (1.17 ∼ 1.54)	<.001		196 (11.6)	1.23 (0.94 ∼ 1.61)	.128		149 (8.8)	1.34 (1.01 ∼ 1.79)	.046	
**Stroke = no**					.773				.446				.64
CysC Tertile 1	652	109 (16.7)	1 (Ref)			24 (3.7)	1 (Ref)			32 (4.9)	1 (Ref)		
CysC Tertile 2	636	231 (36.3)	1.29 (1.01 ∼ 1.64)	.039		57 (9)	1.21 (0.73 ∼ 2)	.451		57 (9)	1.24 (0.78 ∼ 1.97)	.358	
CysC Tertile 3	617	420 (68.1)	1.86 (1.42 ∼ 2.43)	<.001		132 (21.4)	1.8 (1.05 ∼ 3.09)	.034		89 (14.4)	1.81 (1.08 ∼ 3.05)	.025	
Trend test	1905	760 (39.9)	1.38 (1.21 ∼ 1.57)	<.001		213 (11.2)	1.38 (1.06 ∼ 1.79)	.016		178 (9.3)	1.36 (1.05 ∼ 1.77)	.019	
**Stroke = yes**													
CysC Tertile 1	6	3 (50)	1 (Ref)			1 (16.7)	1 (Ref)			0 (0)	1 (Ref)		
CysC Tertile 2	23	15 (65.2)	2.37 (0.37 ∼ 15.07)	.359		5 (21.7)	6 (0.13 ∼ 282.04)	.362		2 (8.7)	—	.987	
CysC Tertile 3	46	41 (89.1)	11.35 (1.47 ∼ 87.33)	.02		12 (26.1)	129.48 (0.68 ∼ 24535.4)	.069		3 (6.5)	—	.992	
Trend test	75	59 (78.7)	4 (1.64 ∼ 9.76)	.002		18 (24)	13.33 (0.93 ∼ 190.81)	.056		5 (6.7)	—	.995	
**Cancer = no**					.352				.137				.303
CysC Tertile 1	622	100 (16.1)	1 (Ref)			21 (3.4)	1 (Ref)			31 (5)	1 (Ref)		
CysC Tertile 2	579	200 (34.5)	1.36 (1.06 ∼ 1.76)	.016		48 (8.3)	1.29 (0.76 ∼ 2.21)	.345		48 (8.3)	1.2 (0.74 ∼ 1.94)	.454	
CysC Tertile 3	540	363 (67.2)	1.9 (1.43 ∼ 2.52)	<.001		119 (22)	2.03 (1.14 ∼ 3.61)	.015		67 (12.4)	1.63 (0.93 ∼ 2.84)	.085	
Trend test	1741	663 (38.1)	1.38 (1.2 ∼ 1.58)	<.001		188 (10.8)	1.46 (1.11 ∼ 1.93)	.007		146 (8.4)	1.28 (0.97 ∼ 1.7)	.078	
**Cancer = yes**													
CysC Tertile 1	36	12 (33.3)	1 (Ref)			4 (11.1)	1 (Ref)			1 (2.8)	1 (Ref)		
CysC Tertile 2	80	46 (57.5)	1.07 (0.51 ∼ 2.24)	.853		14 (17.5)	1.27 (0.33 ∼ 4.84)	.73		11 (13.8)	5.83 (0.59 ∼ 57.84)	.132	
CysC Tertile 3	123	98 (79.7)	2.14 (0.94 ∼ 4.87)	.071		25 (20.3)	1.53 (0.35 ∼ 6.65)	.572		25 (20.3)	18.56 (1.72 ∼ 199.86)	.016	
Trend test	239	156 (65.3)	1.65 (1.12 ∼ 2.43)	.012		43 (18)	1.23 (0.63 ∼ 2.38)	.549		37 (15.5)	3.64 (1.53 ∼ 8.68)	.003	

Adjusted for age, sex, race, marital status, body mass index, education, alcohol user, smoking status, annual family income, eGFR.

Abbreviations: COPD, chronic obstructive pulmonary disease; CVD, cardiovascular disease; CysC, cystatin C; DM, diabetes mellitus; eGFR, estimated glomerular filtration rate; HR, hazard ratio; IFG, impaired fasting glucose.

### The Receiver Operating Characteristic Curves for Serum Biomarkers and Age in Predicting All-Cause, CVD, and Cancer Mortality

**Figure 3.**
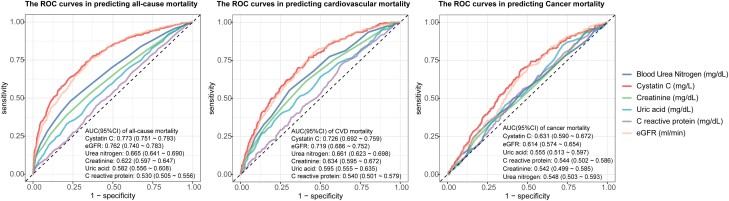
The receiver operating characteristic (ROC) curves for serum biomarkers in predicting all-cause and CVD mortality. (A) The ROC curves for serum biomarkers in predicting all-cause mortality. (B) The ROC curves for serum biomarkers in predicting CVD mortality. (C) The ROC curves for serum biomarkers in predicting cancer mortality.

For the prediction of all-cause mortality among individuals with MetS, the area under the curve (AUC) values (95% CIs) were as follows: CysC, 0.726 (0.692-0.759); eGFR, 0.719 (0.686-0.752); urea nitrogen, 0.661 (0.623-0.698); creatinine, 0.634 (0.595-0.672); uric acid, 0.595 (0.555-0.635); and CRP, 0.540 (0.501-0.579) (Fig. 3A).

For the prediction of CVD mortality, the AUC values (95% CIs) were: CysC, 0.726 (0.692-0.759); eGFR, 0.719 (0.686-0.752); urea nitrogen, 0.661 (0.623-0.698); creatinine, 0.634 (0.595-0.672); uric acid, 0.595 (0.555-0.635); and CRP, 0.540 (0.501-0.579) (Fig. 3B).

Regarding cancer mortality prediction, the AUC values (95% CIs) were: CysC, 0.631 (0.590-0.672); eGFR, 0.614 (0.574-0.654); uric acid, 0.555 (0.513-0.597); CRP, 0.544 (0.502-0.586); creatinine, 0.542 (0.499-0.585); and urea nitrogen: 0.548 (0.503-0.593) (Fig. 3C).

Notably, CysC demonstrated the highest predictive efficacy across all mortality outcomes, followed by eGFR, while urea nitrogen, creatinine, uric acid, and CRP exhibited relatively lower predictive performance for all-cause, CVD, and cancer mortality (Fig. 3C).

**Figure 4.**
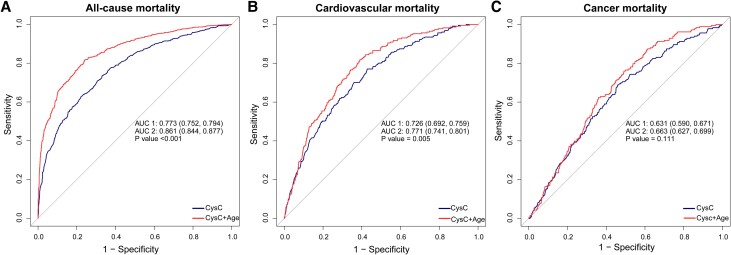
The receiver operating characteristic (ROC) curves for CysC and age in predicting all-cause and CVD mortality. (A) The ROC curves for CysC and age in predicting all-cause mortality. (B) The ROC curves for CysC and age in predicting CVD mortality. (C) The ROC curves for CysC and age in predicting cancer mortality.

Furthermore, the combination of CysC with age significantly enhances the predictive accuracy of mortality. Joint consideration of CysC and age substantially improves the AUC, raising it to 0.861 (95% CI, 0.844-0.877) for all-cause mortality and to 0.771 (95% CI, 0.741-0.801) for CVD mortality (Fig. 4A and 4B, respectively) (*P* < .05). In terms of cancer mortality, the AUC for CysC in predicting cancer mortality is 0.631 (95% CI, 0.590-0.671) (Fig. 4C), while the combined AUC for CysC and age is 0.663 (95% CI 0.627-0.699) (Fig. 4C) (*P* > .05).

## Discussion

Our study has unearthed a compelling association between CysC levels and mortality risks in individuals with MetS. The significance of our findings lies in the revelation of CysC's broader prognostic implications beyond its traditional role in renal function assessment. Notably, the observed dose-dependent relationships between elevated CysC levels and heightened risks of all-cause, CVD, and cancer mortality underscore the multi-dimensional nature of CysC as a prognostic indicator in the context of MetS. The study's results address the unmet need for robust prognostic markers in this at-risk population, underscoring the clinical significance of CysC in risk stratification and prognostication for individuals with MetS.

Our results shed light on the demographic and clinical landscape of individuals with elevated CysC levels within the MetS population. We observed distinct differences in age, gender distribution, race composition, and the prevalence of comorbidities across CysC tertiles. Notably, higher CysC tertiles were associated with altered metabolic parameters, including lower albumin, higher globulin levels, and elevated markers of renal dysfunction (creatinine, uric acid, and blood urea nitrogen). Inconsistent lipid profile trends and marginally significant CRP levels suggest renal impact on lipid metabolism and potential systemic inflammation. Higher CysC tertiles also exhibited a higher burden of chronic conditions, emphasizing the intricate interplay between CysC levels, aging, and the complexity of multimorbidity in this population.

CysC is implicated in the adverse prognosis of several diseases. A cohort study involving 1502 individuals suggests that CysC may exert detrimental effects on metabolism, particularly in the context of abdominal obesity, thereby potentially promoting and predicting the onset and progression of MetS (25). Furthermore, an 8-year follow-up study of 7027 individuals indicates that serum CysC levels are associated with an increased incidence of diabetes and elevated mortality risk in middle-aged and elderly populations (26). Notably, CysC is proposed as a potential biomarker for heightened risks of MetS, CVDs, and renal impairment (27). Additionally, it serves as a marker for muscle wasting (28, 29), a condition linked to increased all-cause mortality, with a causal relationship observed between muscle wasting and overall mortality (30).Our study found a clear dose-response relationship between CysC levels and mortality risk, with higher CysC levels consistently associated with elevated risks of all-cause, CVD, and cancer mortality. The robustness of these associations was further validated through comprehensive covariate adjustments, underscoring the independent prognostic value of CysC as a biomarker for adverse mortality outcomes in individuals with MetS.

This study offers valuable insights into the underlying pathophysiological mechanisms contributing to heightened mortality risks in MetS population. CysC, known to be involved in processes related to inflammation and endothelial dysfunction, serves as a crucial link to the development and progression of CVD and cancer (29, 31). Elevated levels of CysC reflect increased inflammatory burden, insulin resistance, and endothelial dysfunction, providing a mechanistic explanation for the elevated risk of adverse CVD and oncological outcomes (10, 11, 32, 33). Furthermore, impaired renal function, as indicated by elevated CysC levels, mirrors the systemic impact of MetS and contributes to the amplified risk of mortality. Additionally, the close association of CysC with atherosclerosis, a primary pathological process underlying CVD events, enhances its predictive value for CVD mortality (12, 31). These mechanistic insights provide a deeper understanding of the complex interplay between CysC and mortality risk in this population, emphasizing the pivotal role of this biomarker in clinical prognosis and risk assessment.

We found that CysC emerged as the most robust predictor across all-cause and CVD mortality outcomes in patients with MetS, demonstrating superior predictive efficacy compared to eGFR, urea nitrogen, creatinine, uric acid, and CRP. Notably, our analysis also identified eGFR as a significant predictor, albeit exhibiting slightly lower predictive efficacy compared to CysC. The combination of CysC with age substantially enhanced the predictive accuracy for mortality outcomes, particularly for all-cause and CVD mortality. In contrast, urea nitrogen, creatinine, uric acid, and CRP exhibited relatively lower predictive performance across all mortality outcomes. While these biomarkers may provide valuable insights into metabolic and inflammatory processes, their predictive utility for mortality risk assessment in MetS patients appears limited compared with CysC and eGFR. These findings strongly suggest that CysC may emerge as a promising biomarker for assessing the risk of all-cause mortality and CVD death in MetS patients, offering invaluable insights for clinical practice. Integrating CysC assessment into risk stratification protocols may empower clinicians to pinpoint high-risk individuals who stand to benefit from targeted interventions aimed at ameliorating the elevated mortality risks associated with heightened CysC levels. Beyond risk stratification, the incorporation of CysC into clinical practice has the potential to revolutionize therapeutic monitoring and guide personalized interventions.

While our study benefits from a large sample size, a prolonged follow-up duration, and meticulous adjustments for potential confounders, it is important to acknowledge the observational nature of our study, which introduces inherent limitations. Although our study rigorously adjusted for various covariates, residual confounding cannot be entirely excluded. Therefore, future prospective studies and randomized controlled trials are warranted to further validate our findings and investigate the potential therapeutic implications of modulating CysC levels. Additionally, mechanistic studies are needed to unravel the biological underpinnings of the observed associations and to identify potential targets for intervention or risk modification.

In conclusion, our study provides compelling evidence for the significant impact of CysC levels on mortality outcomes in individuals with MetS, shedding light on its potential as a valuable biomarker for risk stratification and prognostication. The integration of CysC assessment into risk stratification frameworks holds promise for refining risk assessment strategies and guiding personalized interventions in individuals with MetS. Moving forward, further research endeavors are essential to harness the full potential of CysC as a prognostic tool and to develop targeted interventions aimed at improving the outcomes of individuals with MetS and elevated CysC levels.

## Data Availability

Publicly available datasets were analyzed in this study. This data can be found at: https://www.cdc.gov/nchs/nhanes/index.htm.

## Abbreviations

AUC: area under the curve
BMI: body mass index
COPD: chronic obstructive pulmonary disease
CRP: C-reactive protein
CVD: cardiovascular disease
CysC: cystatin C
DM: diabetes mellitus
eGFR: estimated glomerular filtration rate
HDL-C: high-density lipoprotein-cholesterol
HR: hazard ratio
MetS: metabolic syndrome
NHANES: National Health and Nutrition Examination Survey
